# The pharmacokinetics of [^18^F]UCB-H revisited in the healthy non-human primate brain

**DOI:** 10.1186/s13550-021-00777-8

**Published:** 2021-04-07

**Authors:** Sébastien Goutal, Martine Guillermier, Guillaume Becker, Mylène Gaudin, Yann Bramoullé, André Luxen, Christian Lemaire, Alain Plenevaux, Eric Salmon, Philippe Hantraye, Olivier Barret, Nadja Van Camp

**Affiliations:** 1 Université Paris-Saclay, CEA, CNRS, MIRCen, Laboratoire Des Maladies Neurodégénératives, 18 Route du Panorama, 92265 Fontenay-aux-Roses, France; 2 GIGA Cyclotron Research Centre In Vivo Imaging, University of Liege, Allee du 6 Aout, 8, Sart Tilman B30, 4000 Liege, Belgium

**Keywords:** SV2A, Cynomolgus non-human Primates, PET, Synaptic imaging

## Abstract

**Background:**

Positron Emission Tomography (PET) imaging of the Synaptic Vesicle glycoprotein (SV) 2A is a new tool to quantify synaptic density. [^18^F]UCB-H was one of the first promising SV2A-ligands to be labelled and used in vivo in rodent and human, while limited information on its pharmacokinetic properties is available in the non-human primate. Here, we evaluate the reliability of the three most commonly used modelling approaches for [^18^F]UCB-H in the non-human cynomolgus primate, adding the coupled fit of the non-displaceable distribution volume (V_ND_) as an alternative approach to improve unstable fit. The results are discussed in the light of the current state of SV2A PET ligands.

**Results:**

[^18^F]UCB-H pharmacokinetic data was optimally fitted with a two-compartment model (2TCM), although the model did not always converge (large total volume of distribution (V_T_) or large uncertainty of the estimate). 2TCM with coupled fit K_1_/k_2_ across brain regions stabilized the quantification, and confirmed a lower specific signal of [^18^F]UCB-H compared to the newest SV2A-ligands. However, the measures of V_ND_ and the influx parameter (K_1_) are similar to what has been reported for other SV2A ligands. These data were reinforced by displacement studies using [^19^F]UCB-H, demonstrating only 50% displacement of the total [^18^F]UCB-H signal at maximal occupancy of SV2A. As previously demonstrated in clinical studies, the graphical method of Logan provided a more robust estimate of V_T_ with only a small bias compared to 2TCM.

**Conclusions:**

Modeling issues with a 2TCM due to a slow component have previously been reported for other SV2A ligands with low specific binding, or after blocking of specific binding. As all SV2A ligands share chemical structural similarities, we hypothesize that this slow binding component is common for all SV2A ligands, but only hampers quantification when specific binding is low.

**Supplementary Information:**

The online version contains supplementary material available at 10.1186/s13550-021-00777-8.

## Background

Synaptic vesicle glycoproteins (SV) are critical to proper nervous system function and have been demonstrated to be involved in vesicle trafficking. They belong to the Major Facilitator Superfamily (MFS) of transporters and consist of a 12-transmembrane glycoprotein and a cytoplasmic N-terminal region containing a long sequence that varies among the three SV2 isoforms (SV2A, SV2B and SV2C) [1, 2]. SV2A and -B are highly homologous to each other, with SV2A showing ubiquitous expression in both excitatory and inhibitory synapses throughout the entire brain [3, 4]; in contrast, SV2B and SV2C are present in a more restricted pattern in the brain, and in only a subset of synapses [2, 5]. The hypothesis of SV2 as vesicular transport protein is based on a significant homology to other transport proteins, however no endogenous substrate has been reported [3], neither has any transport activity been demonstrated [2]. Another hypothetical function is vesicle trafficking and exocytosis, and the modification of the synaptic function [4–6].

Lynch and coworkers identified SV2A as the brain-binding site of the anti-epileptic drug levetiracetam (LEV, Keppra®, UCB Pharma Ltd., Slough, Berkshire, UK) [7]. Seizure protection by LEV and other SV2A ligands strongly correlates with the degree of SV2A occupancy in vivo [3, 8]. However, the site of SV2A-LEV interaction and the mechanism of action remain unclear. LEV does not cause a SV2A conformational state change and it is assumed that SV2A transports LEV or LEV prevents transport of the endogenous substrate [4], as one of the functional consequences of LEV binding to SV2A in brain slices is reduced exocytosis [6]. With the aim of a better understanding of the role of SV2A in epilepsy and of studying SV2A in diseases of the central nervous system, several SV2A-specific ligands have been developed [9], [^18^F]UCB-H being one of the first to be labelled [10], subsequently characterized in the rodent [10, 11], and in the human brain [12]. The demonstration of the co-localization of SV2A with other synaptic markers using [^11^C]-UCB-J [13], showed the potential of in vivo imaging of the synaptic density using Positron Emission Tomography (PET), and led recently to the development of new [^18^F]-labelled ligands [9, 14–19]. Preclinical characterization of [^18^F]UCB-H has mostly been done in the rodent brain [20–22], while in non-human primates (NHP) limited data is available [23, 24]. In humans a preliminary study was performed on four healthy subjects [12], preceding a clinical study in Alzheimer’s patients [25]. Here, we aimed to characterize pharmacodynamics properties of [^18^F]UCB-H in non-human cynomolgus primates in complement to these existing data and to discuss the obtained results in the light of the current state of SV2A PET ligands.

## Material and methods

### Animals

Experiments were conducted on four young adult male cynomolgus monkeys (Macaca fascicularis, 5.2 ± 1.1 kg, 4.4 ± 0.7 years). Animal use procedures were in accordance with the recommendations of the European regulations (EU Directive 2010/63) and approved by the local ethical committee (CETEA n°44), and the French Ministry of Education and Research (NEUROMODEL: APAFIS#389-20150327162135690v02). The experimental data reported in this study are in compliance with the ARRIVE (Animal Research: Reporting in Vivo Experiments) guidelines [26].

### Radiochemistry

Radiosynthesis of the enantiomeric ligand [^18^F]UCB-H was realized through a one-step radiolabelling of a pyridyliodonium precursor as previously described [27]. [^18^F]UCB-H was formulated in 0.9% aqueous saline with 3% ethanol (v/v). The radiochemical purity of [^18^F]UCB-H was > 98% and the molar activity at the time of injection was 54 ± 32 GBq/μmol.

### PET imaging

#### Experimental design

All four non-human primates (NHP) underwent a 2-h (2 h) test and retest PET scan; two NHP underwent an additional 4-h (4 h) retest PET scan. Three NHP underwent one 4-h (4 h) displacement PET scan. Arterial blood sampling was performed during test and retest scans with PET imaging spaced by at least 3 weeks between each scan. To this end, the femoral artery opposite to the saphenous vein used for radioligand injection was cannulated. For the 2-h (2 h) scans, we collected in total 28 blood samples of 1 mL: 16 samples during the first 5 min followed by 3 samples every 5 min and 11 samples every 10 min. For the 4 h scans, we collected an additional 6 samples every 20 min during the last two hours (34 samples in total). Larger samples (2 – 3 mL) were collected at 5, 15, 30, 60, 90, 120 min (2 h) and at 180, 240 min (4 h) for metabolite analysis. Displacement of [^18^F]UCB-H was done by intravenous (IV) administration 90 min after [^18^F]UCB-H injection of a bolus of 30 mg/kg LEV, or of 80 µg/kg (≈250 nmol/kg) or 5 mg/kg (≈15 µmol/kg) of cold [^19^F]-UCB-H. Full experimental design of all NHP is outlined in Additional file 1: Table 1.

#### Drug formulation

Solvents and LEV (C_8_H_14_N_2_O_2_; MM 170.21 g/mol) were obtained at Sigma-Aldrich® (France), and [^19^F]-UCB-H (C_16_H_12_F_4_N_2_O; MM 324.28 g/mol) was synthesised as previously described [27]. Injectable solutions of [^19^F]-UCB-H for displacement studies were prepared with a mixture of Tetrahydrofuran (THF), Médialipide® and glucose at 2.5% as previously described [28]. LEV was dissolved in a glucose solution of 2.5% to reach a concentration of 90 mg/mL.

#### PET imaging

PET imaging was performed on the microPET FOCUS220 (Siemens) under standard anesthesia and monitoring procedures [29]. Data acquisition started with the IV bolus injection of [^18^F]UCB-H (32.9 ± 1.0 MBq/kg, 0.35 ± 0.07 µg/kg). Dynamic PET images were reconstructed using standard OSEM-2D algorithms while correcting for radioactive decay, scatter, attenuation and detectors inhomogeneity, which were measured prior to PET scanning using respectively ^57^Co and ^68^Ge external sources.

#### Blood measurement & analysis

Plasma was separated from whole-blood by centrifugation (5 min, 2054x*g*, 4 °C) and 50 µL of plasma and whole-blood were counted using a PET cross-calibrated gamma well counter (WIZARD^2^, PerkinElmer, France) to obtain the whole-blood and plasma activity curves. All data were corrected for radioactive decay from the injection time. For the larger blood samples, 500 µL plasma was deproteinized with acetonitrile. The supernatant was injected in high-performance liquid chromatography, equipped with an Atlantis® T3 5 μm 4.6 × 150 mm column (Waters) and an Atlantis® T3 5 μm 3.9 × 5 mm pre-column (Waters), with an LB-513 radioactivity flow detector (Berthold, La Garenne Colombes, France, MX Z100 cell). The eluant was collected in intervals of 15 s (fraction collector III, Waters, France) and counted in the gamma well counter (WIZARD^2^, PerkinElmer, France) to measure total activity. [^18^F]UCB-H parent fraction was calculated as a percentage of the total radioactivity (metabolites and parent).

For each animal, a 2-exponential decay function was fitted to [^18^F]UCB-H parent fraction, which was time multiplied with the plasma activity curve to obtain the metabolite-corrected arterial plasma input function (mcAIF) used for the kinetic modeling.

The fraction of [^18^F]UCB-H in NHP plasma samples not bound to plasma protein was measured before PET injection using a previously described ultrafiltration method [30]. In brief, standard amounts of [^18^F]UCB-H (≈15 kBq) were added to 200 μL plasma that was applied to Microcon® filtration devices containing an YM-10 membrane (Millipore, France). The devices were centrifuged for 10 min at 10,000 g (Sigma 2-16KL, France). [^18^F]UCB-H activity concentration in the resulting ultrafiltrate (≈70 μL, CFP) and a sample of plasma (CP) were counted. The free fraction (f_p_) was calculated as: f_p_ = CFP/CP and measured in triplicates.

#### PET data analysis

PET image analysis was performed using PMOD software version 3.8 (PMOD Technologies Ltd., Zurich, Suisse). After individual co-registration of PET-MR images, a cynomolgus atlas published by Ballanger and coworkers [31] was normalized to PET images to extract time activity curves in different brain regions. Volumes of interest (VOI) were cerebral white matter (16.7 cm^3^), striatum (0.24 cm^3^), thalamus (0.89 cm^3^), cerebellum (3.83 cm^3^), frontal—(2.50 cm^3^), parietal—(5.35 cm^3^), and temporal (8.22 cm^3^) cortex, and whole brain (72.2 cm^3^) as a composite region of all regions in the atlas. [^18^F]UCB-H pharmacokinetics were evaluated by analyzing the time activity curves of the test- and retest scans using 1- and 2-tissue compartment models (unconstrained and constrained with global K_1_/k_2_ coupled fit across all regions, further referred to as 2TCM and 2TCM-c) [32], and Logan graphical analysis with a fixed t* at 60 min [33] using the arterial plasma input function corrected for radio-metabolites to derive the volume of distribution in each region. Percentage standard error (%SE) was estimated from the theoretical parameter covariance matrix. Only reliable estimates (%SE less than 25%) were included in the current analyses. For the three drug studies, percent displacement of the total activity in the whole brain was estimated relative to an average baseline constructed from the two 4 h baseline studies. Briefly, Standardized Uptake Value (SUV) time activity curves (TACs) were calculated for the two baseline and the three displacement studies. Then, each curve was normalized to the SUV at t = 90 min, corresponding to the timeframe immediately before the administration of the drugs in the displacement studies. Baseline curves were averaged and the percent displacement of total activity was calculated as (baseline-displacement)/baseline at a given time post drug administration (see “Results”).

#### Statistical analysis

Statistical analysis was performed using R software (version 3.3.1.). Plasma, intact parent fraction and time activity curves were statistically compared between subjects using a one-factor variance analysis. *Absolute Test–Retest variability* (aTRV) of PET quantification parameters was calculated as ABS(test—retest) / AVERAGE(test, retest). *Reliability* was evaluated using a two-factor mixed model in order to calculate the intra-class correlation coefficient (ICC) (Package Psych, Version 1.7.8). All values are expressed as average ± standard error of the mean (SEM; significance level was fixed at *p* < 0.05).

## Results

### Blood data

**Fig. 1:**
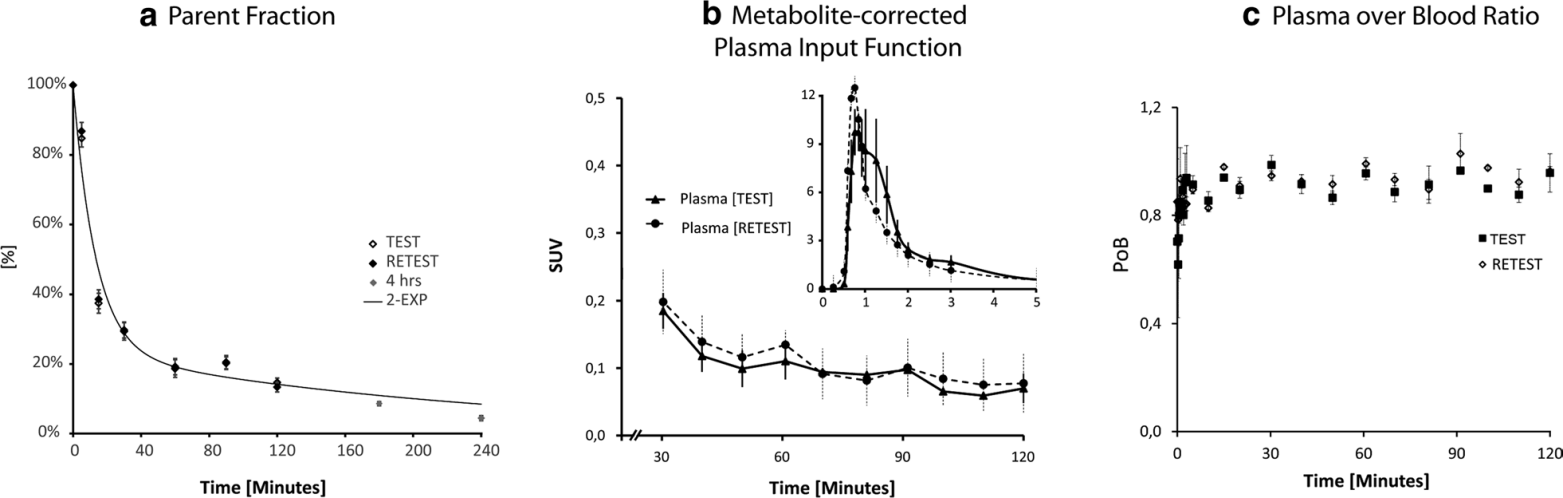
Blood measures after i.v. [^18^F]UCB-H injection in the cynomolgus NHP in test- and retest studies. Parental fraction (in %) was fitted with a two-exponential decay function based on blood data acquired up to 4 h after tracer injection (**a**). Average (± SEM) metabolite-corrected plasma input function (**b**) and plasma to whole blood ratio (**c**) were highly consistent between and within animals

[^18^F]UCB-H shows a rapid metabolism in the arterial blood with an intact parent fraction of 37 ± 3.9% at 15 min, 29.5 ± 3.4% at 30 min, 19 ± 3.5% at 60 min, 14.6 ± 1.9% at 120 min and 4.4 ± 0.7% at 240 min after tracer injection. The parent fraction was fitted with a two-exponential decay function based on blood data acquired up to 4 h after tracer injection, and on the residuals (Fig. 1a). Whole blood and plasma-input functions were highly consistent between animals with stable plasma to whole-blood ratio of 0.90 ± 0.05 over the entire 120-min acquisition period (Fig. 1b, c). Plasma free parent fraction (*f*_p_) measured by ultrafiltration before tracer injection was 42.6 ± 1.6%.

### Brain kinetics

#### Time activity curves

**Fig. 2:**
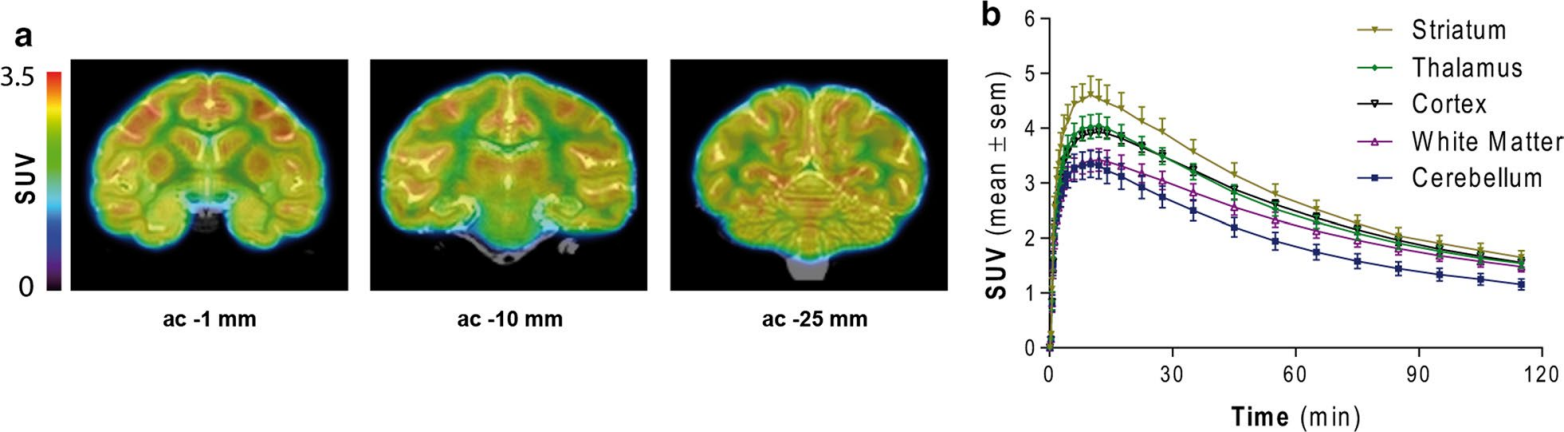
**a** Representative 60–120 min summed coronal PET images at ac -1 mm, ac – 10 mm and ac -25 mm coregistered to T_2_-weighted MR images. **b** Average (± SEM) TAC in striatum, thalamus, cortex, white matter and cerebellum

Figure 2b shows representative time-activity curves (TACs) in a subset of brain regions. [^18^F]UCB-H entered rapidly in the brain, reaching maximal uptake 5 to 15 min after injection, followed by a relatively slow terminal elimination phase. Highest SUV uptake was observed in the striatum, followed by the thalamus and the cortical regions.

#### Compartment modeling

**Fig. 3:**
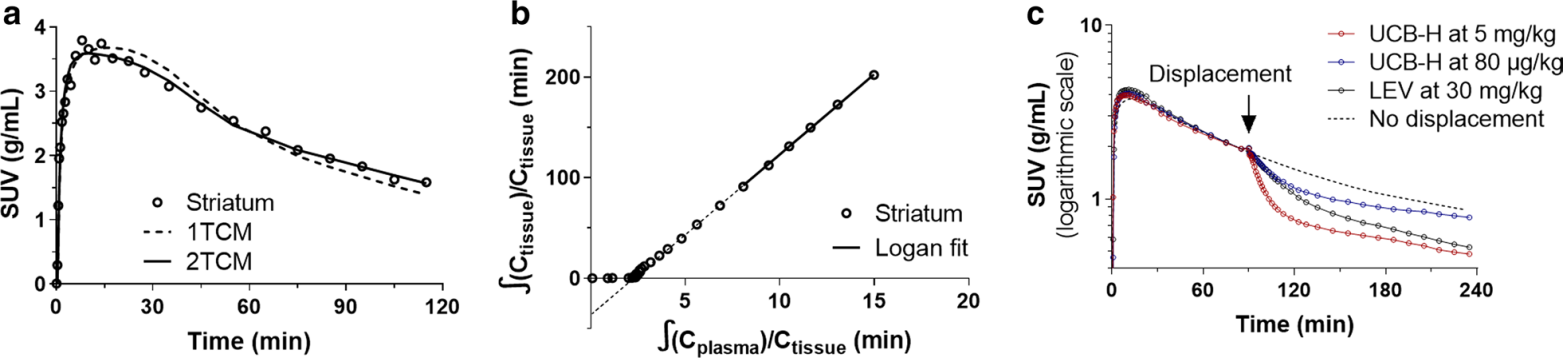
**a** According to the lowest Akaike Information Criterion (AIC), the two-compartment model (2TCM, full line) fitted better the TACs (here striatum, circles) compared to the one-compartment model (1TCM, dashed line). **b** Logan graphical linear fit of striatal TAC (circles) with t* fixed at 60 min. **c** Dose dependent displacement of [^18^F]UCB-H at 90 min after radioligand injection by [^19^F]UCB-H at 80 µg/kg (red) and 5 mg/kg (blue). Slower brain penetrance of LEV (30 mg/kg, black) induced a slower displacement

According to the lowest Akaike Information Criterion (AIC), the two-compartment model (2TCM) fitted better the TACs compared to the one-compartment model (1TCM) (AIC_1TCM_ = 21.9 ± 1.8 *vs*. AIC_2TCM_ = 0.71 ± 1.73). However, across all scans and VOI, V_T_ estimated by 2TCM was only quantifiable (%SE < 25%) in 50% of cases (Fig. 3a and Additional file 1: Fig. 1). There was high uncertainty on the estimation of k_3_ and even higher for k_4_ where the estimate tended toward 0 in 30% of cases independent of the VOI, with therefore unreliable estimates of k_3_/k_4_ in > 80% of cases. 2TCM-c was more stable and enabled estimation of V_T_ in 80% of cases, and of k_3_/k_4_ in 70% of cases. Logan graphical analysis fitted robustly all TACs, with a representative plot shown in Fig. 3b.

**Fig. 4:**
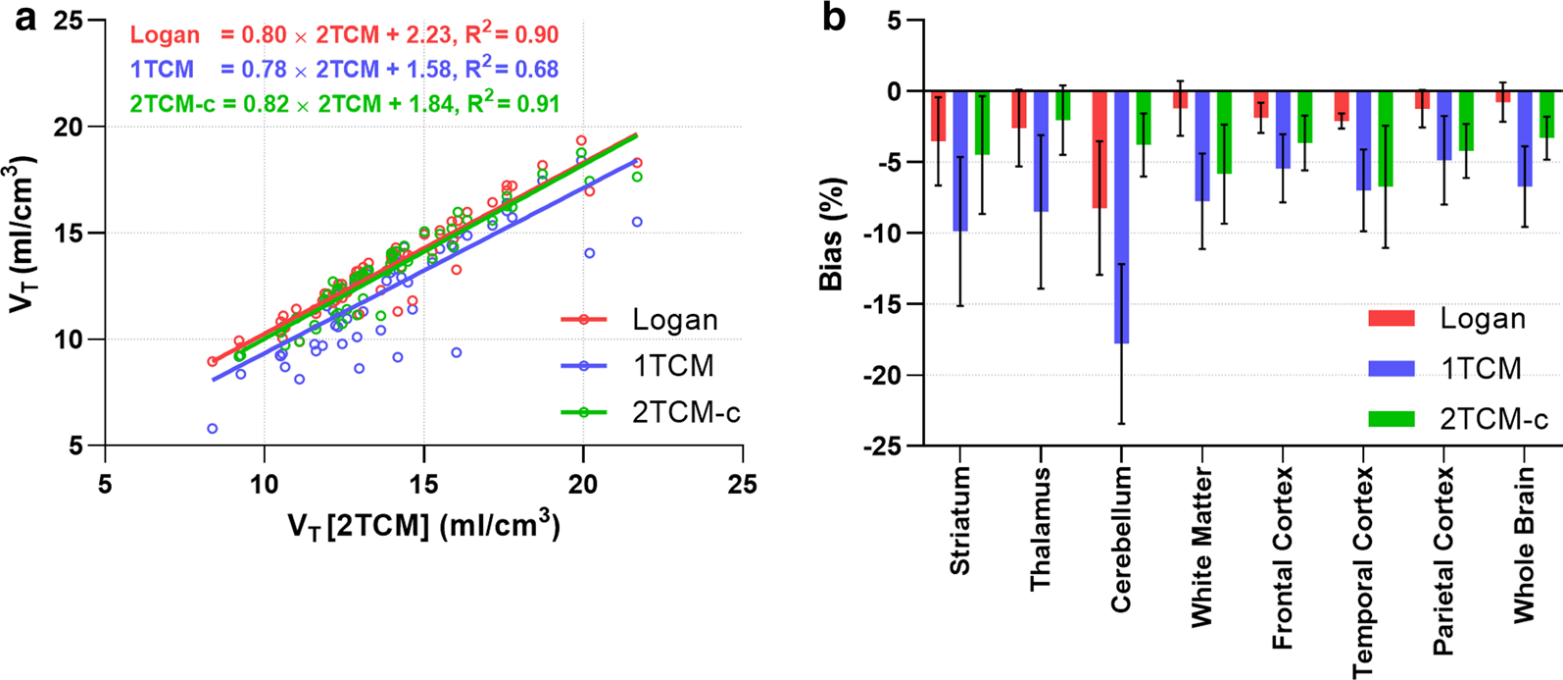
**a** Linear correlations show a better agreement between 2TCM, Logan and 2TCM-c as compared to 1TCM. **b** Overall, Logan and 2TCM-c provide lower underestimation of V_T_ (-3 ± 1% and -4 ± 1% respectively), as compared to 1TCM (-9 ± 1%)

Figure 4a shows correlation plots between V_T_ estimates obtained with Logan, 1TCM, 2TCM-c, and the cases for which 2TCM converged. Good agreement was found between 2TCM and Logan or 2TCM-c estimates (R^2^ = 0.90 and R^2^ = 0.91, respectively) with a slight overall underestimation of V_T_ (-3 ± 1% for Logan, and -4 ± 1% for 2TCM-c), while 1TCM had a moderate correlation with 2TCM (R^2^ = 0.68) and overall larger bias for V_T_ (-9 ± 1%) (Fig. 4b).

A summary of V_T_ obtained by the different methods (2TCM, 2TCM-c, 1TCM and Logan) for different regions is shown in Table 1. aTRV and ICC were calculated for V_T_ obtained by 2TCM-c, and Logan. Overall, aTRV were lower, and ICC were higher for Logan (~ 11% and ~ 0.6) compared to 2TCM-c (~ 17% and ~ 0.4) (Table 2).

**Table 1:** Regional estimates of V_T_- and BP_ND_ for test-, and retest scans

Brain region	2TCM			2TCM-c			1TCM			Logan (t* = 60)			2TCM-c		
	V_T_ [mL/cm^3^]			V_T_ [mL/cm^3^]			V_T_ [mL/cm^3^]			V_T_ [mL/cm^3^]			k_3_/k_4_ (BP_ND_)		
	Mean	±	SEM	Mean	±	SEM	Mean	±	SEM	Mean	±	SEM	Mean	±	SEM
Striatum															
Test	15.3	±	2.25	15.0	±	1.11	14.2	±	1.29	15.8	±	1.15	1.16	±	0.36
Retest	14.1	±	0.96	15.3	±	0.93	14.8	±	0.61	15.7	±	1.04	1.12	±	0.39
Thalamus															
Test	15.6	±	1.98	15.0	±	1.38	13.3	±	1.17	14.9	±	0.99	1.03	±	0.25
Retest	13.0	±	1.08	14.2	±	1.06	13.4	±	0.60	14.3	±	1.11	0.97	±	0.36
Cerebellum															
Test	10.6	±	0.08	9.70	±	*NA*	9.8	±	0.78	11.2	±	0.68	1.24	±	*NA*
Retest	12.3	±	*NA*	13.3	±	2.97	10.4	±	0.82	11.1	±	0.75	1.22	±	0.42
White matter															
Test	14.0	±	2.37	13.8	±	1.69	12.3	±	1.13	13.9	±	1.00	1.25	±	0.16
Retest	12.6	±	1.08	13.7	±	1.61	12.5	±	0.68	13.6	±	1.23	0.83	±	0.47
Frontal cortex															
Test	16.3	±	3.68	14.8	±	2.15	13.8	±	1.64	15.0	±	1.59	1.25	±	0.32
Retest	13.1	±	0.66	14.7	±	1.37	13.8	±	1.15	14.8	±	1.87	1.32	±	0.40
Temporal cortex															
Test	13.9	±	1.60	14.8	±	1.30	12.9	±	0.83	14.1	±	0.77	1.00	±	0.24
Retest	14.0	±	0.01	13.1	±	0.76	13.0	±	0.52	13.9	±	0.91	0.97	±	0.54
Parietal cortex															
Test	15.5	±	3.24	15.4	±	1.54	14.3	±	1.41	15.4	±	1.35	1.08	±	0.26
Retest	13.6	±	0.36	16.1	±	1.40	14.8	±	0.75	15.7	±	1.43	1.17	±	0.29

V_T_ was estimated using 2TCM, 2TCM-c, 1TCM and Logan. BP_ND_ was estimated by k_3_/k_4_ using a 2TCM-c. Only estimates at < 25%SE were included. NA = undetermined

**Table 2:** Summary of aTRV and ICC values for V_T_ estimates by 2TCM-c and Logan

Brain region	2TCM-c V_T_ (mL/cm^3^)		Logan (t* = 60) V_T_ (mL/cm^3^)	
	aTRV	ICC	aTRV	ICC
Striatum	12%	0,52	11%	0.67
Thalamus	18%	0,05	12%	0.61
Cerebellum	*NA*	*NA*	14%	0.32
White matter	24%	0.43	13%	0.62
Frontal cortex	11%	0.84	11%	0.89
Temporal cortex	25%	*NA*	13%	0.61
Parietal cortex	19%	0.38	12%	0.75

aTRV and ICC calculation of the most relevant V_T_ estimates obtained with 2TCM-c and Logan. Only estimates at <25%SE were included. For 2TCM, only NHP2 had acceptable estimates in both test-, and retest conditions.

As surrogate of BP_ND_, k_3_/k_4_ obtained by 2TCM-c, was estimated to be 1.07 ± 0.02 on average; regional test, and retest values are detailed in Table 1. The tissue influx parameter of [^18^F]UCB-H (K_1_), a measure of blood flow and tracer extraction, was equal for 2TCM and 2TCM-c and estimated to 0.37 ± 0.01 mL/cm^3^/min in grey matter regions. The average V_ND_ (K_1_/k_2_ from 2TCM-c) over all VOIs and animals was 7.89 ± 1.23 mL/cm^3^, with a free fraction in tissue f_ND_ of 6.1 ± 1.3%. Additional file 1: Table 2 summarizes all individual estimates (< 25%SE) by 2TCM, 2TCM-c, 1TCM and Logan.

### Displacement studies

In all brain regions, [^18^F]UCB-H uptake was displaced by [^19^F]UCB-H in a dose-dependent manner with the displacement occurring rapidly; brain penetration of [^19^F]UCB-H is fast with maximum uptake 5–15 min after injection. Low dose of [^19^F]UCB-H (80 µg/kg, more than 100 fold [^18^F]UCB-H mass dose) resulted in ~ 20–25% displacement, and high dose of [^19^F]UCB-H (5 mg/kg) displaced close to 50% of [^18^F]UCB-H, where both measurements were estimated at 35 min after injection of cold [^19^F]UCB-H when the displacement was maximum (Fig. 3c).

LEV administration at pharmacological dose has a much slower brain penetration [34], and a dose of 30 mg/kg induced a slower displacement of [^18^F]UCB-H of ~ 40% as estimated at 150 min after injection of LEV (at the end of the 240 min acquisition) (Fig. 3c).

## Discussion

The current study evaluates [^18^F]UCB-H pharmacokinetics in healthy NHPs. We demonstrated similar metabolism in NHP as previously described in the rodent, human and rhesus monkey [11, 12, 23]. We have shown that [^18^F]UCB-H equilibrates rapidly between whole blood and plasma, with high availability in plasma (*f*_p_ of 42.6 ± 1.6%). Similar to data obtained in rodent and human [11, 12], we observed in the NHP a good brain penetrance with ubiquitous brain uptake. Tissue influx parameters of [^18^F]UCB-H were comparable to other SV2A radioligands for grey matter regions [13, 24]. Even though a better goodness of fit of the TACs was obtained with 2TCM compared to 1TCM, not all TACs could be fitted with 2TCM. Previously, this has been the criteria to preconize the graphical method of Logan [12]. Here we demonstrate that Logan provides reliable estimates of V_T_ with a relatively low aTRV (~ 12%), although larger than that reported for [^11^C]UCB-J in humans (~ 4%)[35], and a small bias relative to 2TCM of ~ -3% compared to ~ -9% for 1TCM. In a preliminary rhesus NHP study, Zheng and colleagues alternatively proposed 1TCM and the multilinear analysis method (MA1) to obtain reliable estimates of V_T_ for [^18^F]UCB-H [23]. Here, in the cynomolgus NHP, we demonstrated an aTRV above 10% for 1TCM and a poor reliability. For MA1 we found aTRV of V_T_ (~ 11%), but a larger negative bias (~ -8%) compared to Logan (*data not shown*).

We observed that the convergence issue with 2TCM was mainly due to instability of the k_4_ estimate tending to zero in ~ 30% of all cases, independent of the regions. Brain kinetics of SV2A ligands [^11^C]UCB-J [35, 36] and [^18^F]SynVesT-1 (a.k.a [^18^F]SDM-8, [^18^F]MNI-1126) [14] have been reported as better described by 2TCM in human, but were better modeled with 1TCM in cynomolgus or rhesus monkeys [24, 37, 38], and [^11^C]UCB-A was better described with 1TCM at baseline in pigs but 2TCM was required after blocking of the specific signal [39]. Interestingly, all authors reported issues with 2TCM similar to those reported here, namely lack of convergence or large standard error for V_T_ [14, 35, 36, 39], with large uncertainty on k_4_ and values close to 0 [14, 35]. As a consequence, 1TCM for [^11^C]UCB-J and [^18^F]SynVesT-1 [14, 35], and Logan graphical analysis for [^11^C]UCB-A [39] was selected as method of choice. We reported here similar issues with the use of 2TCM for [^18^F]UCB-H. We obtained similar AIC for reversible or irreversible 2TCM, and spectral analysis indicated a component at the edge of the frequency range (data not shown), suggesting a small slow component for [^18^F]UCB-H. Although this component could reflect penetrating metabolites, inaccuracy of metabolite correction or vascularity activity correction, its exact nature remains unclear. Given the similarity in chemical structures between the current SV2A ligands and the aforementioned results reported in the literature, we hypothesize that this slow component is likely common between all these ligands. Moreover, this component is problematic when the specific signal is low (low affinity ligands or blocking studies) and when kinetics can only be described with 2TCM. This was nicely illustrated with [^18^F]MNI-1126 (high affinity of the (*R*)-enantiomer), [^18^F]MNI-1038 (racemate) and [^18^F]MNI-1128 (low affinity of the (*S*)-enantiomer) [24, 40], where 1TCM was the best model for [^18^F]MNI-1126, but 2TCM had to be used both for [^18^F]MNI-1038 and [^18^F]MNI-1128 (lower specific signal), with very low k_4_ for [^18^F]MNI-1128 [24]. Here, similarly to [^11^C]UCB-A [39], the method of choice for [^18^F]UCB-H was Logan graphical analysis, as 1TCM was not adequate due to its lower affinity compared to other SV2A radioligands.

We performed homologous (with [^19^F]UCB-H) and heterologous (with reference compound LEV) displacement studies to evaluate the reversibility of [^18^F]UCB-H binding. The total uptake of [^18^F]UCB-H was clearly displaceable, and in a dose-dependent manner by [^19^F]UCB-H up to 50% of the total uptake at the highest dose tested (5 mg/kg). Based on [^18^F]UCB-H brain uptake curves and an f_ND_ of ~ 6%, we have a maximum free concentration of UCB-H of ~ 2.5 mM after administration 5.0 mg/kg. For UCB-H, in vitro K_i_ of 9 nM in human brain [40] and in vivo K_D_ of 30 nM in NHP [38] were reported, and conservatively considering a *K*_*D*_ of 30 nM, near full saturation of SV2A would be expected (> 98% occupancy) at a dose of 5 mg/kg and a free concentration of 2.5 mM. We have reported a maximum displacement of the total uptake of 50% with UCB-H, indicating that only about half of the total uptake is displaceable for [^18^F]UCB-H; therefore, BP_ND_ would be expected close to 1.0. Although imperfect because of all the microparameters correlations, k_3_/k_4_ from 2TCM with coupled fit K_1_/k_2_ was taken here as a surrogate of BP_ND_ to estimate the size of the specific signal and compare to previously reported results with other ligands. The expected BP_ND_ close to 1.0 is in agreement with the average value for k_3_/k_4_ of 1.1 reported in Table 1. Lower BP_ND_ values were reported in humans [12], however the binding potentials were derived relative to the centrum semiovale and would likely be higher if calculated using the true V_ND_ [14]. Finally, the displacement study with LEV evidenced its slower brain penetrance compared to UCB-H, with a displacement of the total uptake of ~ 40% measured 2.5 h after 30 mg/kg i.v. LEV administration, which would correspond to an occupancy of ~ 75–80% of SV2A in agreement with values in the literature [14, 24, 38]. We observed some displacement in the white matter both with [^19^F]UCB-H and LEV. This is likely due to high spill-in from cortical regions rather than true specific signal in the white matter, as indicated by the higher than expected uptake and V_T_ [12]. Here, we coregistered T_2_-weighted MR-PET images to the Ballanger template [31] and used the inverse transformation to extract TACs from the PET images. Cynomolgus brains have relatively small white matter regions, with some inter-animal variability. Therefore, this approach appeared less precise to segment white matter regions, and prone to partial volume effect; as such, the current data set did not allow to use or evaluate the utility of the white matter as a reference region.

Altogether, our data confirm similar brain penetration and non-displaceable uptake for [^18^F]UCB-H as described for [^11^C]UCB-J [38], [^18^F]MNI1126 [16] (aka [^18^F]Synvest-1 [14]). In agreement with previous reports in rhesus NHP [23, 38], V_T_-estimates and consequently BP_ND_ of [^18^F]UCB-H in cynomolgus NHP are considerably lower (~ 50% lower for V_T_, and 3 to fourfold lower for BP_ND_) compared to V_T_ and BP_ND_ measures with [^11^C]UCB-J [35, 38] and other [^18^F]-labelled SV2A radioligands, [^18^F]synvest1 [14, 16] and [^18^F]synvest2 [17] (alias [^18^F]SDM-2 [18]). As a consequence, [^18^F]UCB-H will be less sensitive to detect small changes compared to the latest SV2A ligands. Nevertheless, a clinical study using [^18^F]UCB-H in Alzheimer’s Disease (AD) patients demonstrated a correlation between lower synaptic density and poorer awareness of memory functioning in Aβ-positive individuals [25], confirming earlier data in AD patients using [^11^C]UCB-J [41]. Additionally, this [^18^F]UCB-H clinical study [25] suggested a widespread synaptic decline in AD patients across the neocortex and in some subcortical nuclei, including the basal forebrain, which was confirmed in a comparable but larger cohort of AD patients using [^11^C]UCB-J [42]. These and other data [43] have demonstrated the potential of the [^18^F]UCB-H radioligand.

## Conclusions

[^18^F]UCB-H was the first [^18^F]-labeled SV2A radioligand [10, 11], before a long series of new [^18^F]-labeled SV2A candidate ligands [17, 18, 24]. Here, we aimed to complete existing data on [^18^F]UCB-H by pharmacokinetic studies in young NHP. We show that, when specific binding is low, a slow component gives rise to compartment modeling difficulties with 2TCM due to instability of k_4_ estimation, which in 30% of all cases tends to 0. A similar observation has previously been reported for other SV2A candidate ligands [24, 39]. Graphical analysis allows nevertheless a reliable quantification of V_T_ with acceptable aTRV and bias. Despite a lower sensitivity due to lower *BP*_ND_, [^18^F]UCB-H recently provided sound data in a clinical AD study [25], paving the road for synaptic PET imaging using highly specific SV2A ligands in neurodegenerative disorders.

## Supplementary Information

**Additional file 1:** Additional data on experimental design and individual measures.

## Data Availability

The datasets used and/or analyzed during the current study are available from the corresponding author on reasonable request.

## Abbreviations

1TCM: One-compartment model
2TCM: Two-compartment model
%SE: Percentage standard error
AIC: Akaike Information Criterion
aTRV: Absolute Test–Retest variability
BP_ND_: Non-Displaceable Binding Potential
f_p_: Plasma free parent fraction
LEV: Levetiracetam
mcAIF: Metabolite-corrected arterial plasma input function
NHP: Non-human Primates
PET: Positron Emission Tomography
TAC: Time-activity curves
SEM: Standard error of the mean
SUV: Standardized uptake value
VOI: Volumes of interest
V_T_: Total Volume of Distribution
SV2A: Synaptic vesicle glycoprotein 2A
