# Supplementary material for: The pharmacokinetics of [18F]UCB-H revisited in the healthy non-human primate brain
Source: EJNMMI Res. 2021 Apr 7;11:36. doi: 10.1186/s13550-021-00777-8 (PMC8026785; doi:10.1186/s13550-021-00777-8)
Supplement: Supplementary file 1 — Additional file 1: Additional data on experimental design and individual measures. [file 13550_2021_777_MOESM1_ESM.docx]

# Supplementary data

## Supplementary Table 1: Full experimental design of all NHP

| **NHP** | **Protocol** | **Acquisition** | **BW  [kg]** | **ID [MBq]** | **RAS_inj_ [GBq/µmol]** | **Mass [nmol]** | **Weight [µg]** | **Drug** | **Dose [µg/kg]** |
| --- | --- | --- | --- | --- | --- | --- | --- | --- | --- |
| NHP1 | Test | 2 hrs | 3.96 | 122 | 120.6 | 1.01 | 0.33 |  |  |
| NHP2 | Test | 2 hrs | 4.35 | 135 | 103.7 | 1.30 | 0.42 |  |  |
| NHP3 | Test | 2 hrs | 4.30 | 154 | 58.8 | 2.62 | 0.85 |  |  |
| NHP4 | Test | 2 hrs | 4.05 | 142 | 63.0 | 2.26 | 0.73 |  |  |
| NHP1 | Retest | 2 hrs | 4.20 | 154 | 74.0 | 1.73 | 0.56 |  |  |
| NHP2 | Retest | 2 hrs | 4.45 | 129 | 67.7 | 1.59 | 0.52 |  |  |
| NHP3 | Retest | 2 hrs | 4.72 | 173 | 54.1 | 3.42 | 1.11 |  |  |
| NHP4 | Retest | 2 hrs | 5.05 | 163 | 59.1 | 2.96 | 0.96 |  |  |
| NHP3 | Baseline | 4 hrs | 5.47 | 201 | 40.3 | 8.43 | 2.73 |  |  |
| NHP4 | Baseline | 4 hrs | 6.91 | 243 | 43.3 | 9.49 | 3.08 |  |  |
| NHP1 | Displacement | 4 hrs | 5.3 | 194 | 13.7 | 15.75 | 5.11 | LEV | 30000 |
| NHP2 | Displacement | 4 hrs | 6.9 | 205 | 14.4 | 15.79 | 5.12 | UCB_H | 5000 |
| NHP4 | Displacement | 4 hrs | 7.05 | 178 | 18.2 | 9.77 | 3.17 | UCB_H | 80 |

*Abbreviations: BW, Body Weight; ID, Injected Dose, RAS_inj_, Radioactive Specificity at time of injection.*

## Supplementary Table 2: Individual quantitative measures

|  |  |  | **2TCM** | | | | **2TCM-c** | | | | **1TCM** | **Logan** |
| --- | --- | --- | --- | --- | --- | --- | --- | --- | --- | --- | --- | --- |
| **NHP** | **Protocol** | **Voi** | **K_1_ [ml/cm^3^**  **/min]** | **V_T_ [ml/cm^3^]** | **K_1_/k_2_ [ml/cm^3^]** | **k_3_/k_4_** | **K_1_ [ml/cm^3^**  **/min]** | **V_T_ [ml/cm^3^]** | **K_1_/k_2_ [ml/cm^3^]** | **k_3_/k_4_** | **V_T_ [ml/cm^3^]** | **V_T_ [ml/cm3]** |
| **NHP1** | Test | Cerebellum | 0.17 | *NA* | 6.69 | *NA* | 0.17 | *NA* | 7.86 | *NA* | 9.02 | 10.85 |
|  | Retest |  | 0.36 | *NA* | 8.77 | *NA* | 0.36 | 8.78 | 7.25 | *NA* | 8.78 | 8.96 |
|  | Test | Frontal Cortex | 0.22 | *NA* | 11.87 | *NA* | 0.22 | 14.10 | 7.86 | 0.61 | 13.68 | 15.28 |
|  | Retest |  | 0.37 | 12.88 | *NA* | *NA* | 0.37 | 12.90 | 7.25 | 0.78 | 12.88 | 13.18 |
|  | Test | Parietal Cortex | 0.18 | *NA* | 13.88 | *NA* | 0.18 | 17.47 | 7.86 | 1.00 | 15.09 | 16.49 |
|  | Retest |  | 0.33 | 13.26 | *NA* | *NA* | 0.34 | 13.26 | 7.25 | 0.83 | 13.26 | 13.60 |
|  | Test | Striatum | 0.24 | *NA* | 10.32 | *NA* | 0.24 | 14.78 | 7.86 | *NA* | 12.87 | 15.21 |
|  | Retest |  | 0.41 | 13.09 | *NA* | *NA* | 0.42 | 13.08 | 7.25 | 0.80 | 13.04 | 13.39 |
|  | Test | Temporal Cortex | 0.17 | *NA* | 11.10 | *NA* | 0.17 | 17.46 | 7.86 | 1.00 | 12.66 | 14.13 |
|  | Retest |  | 0.31 | *NA* | 11.50 | *NA* | 0.32 | 11.57 | 7.25 | 0.60 | 11.57 | 11.91 |
|  | Test | Thalamus | 0.21 | *NA* | 11.03 | *NA* | 0.20 | 17.47 | 7.86 | 1.00 | 12.88 | 14.58 |
|  | Retest |  | 0.36 | 11.88 | *NA* | *NA* | 0.37 | 11.88 | 7.25 | 0.64 | 11.85 | 12.16 |
|  | Test | White Matter | 0.17 | *NA* | 9.57 | *NA* | 0.17 | 15.44 | 7.86 | *NA* | 12.00 | 14.19 |
|  | Retest |  | 0.29 | 10.58 | *NA* | *NA* | 0.30 | 10.59 | 7.25 | 0.46 | 10.59 | 11.11 |
| **NHP2** | Test | Cerebellum | 0.22 | 10.64 | 5.68 | 0.87 | 0.23 | 9.72 | 4.35 | 1.24 | 8.70 | 10.54 |
|  | Retest |  | 0.62 | 12.28 | *NA* | *NA* | 0.58 | 12.24 | 4.64 | 1.64 | 12.10 | 12.38 |
|  | Test | Frontal Cortex | 0.24 | 12.58 | 8.05 | 0.56 | 0.25 | 11.41 | 4.35 | 1.63 | 10.97 | 12.20 |
|  | Retest |  | 0.45 | 14.38 | *NA* | *NA* | 0.48 | 14.39 | 4.64 | 2.10 | 14.34 | 14.05 |
|  | Test | Parietal Cortex | 0.22 | 12.22 | 7.68 | 0.59 | 0.23 | 11.12 | 4.35 | 1.56 | 10.65 | 11.83 |
|  | Retest |  | 0.42 | 13.98 | *NA* | *NA* | 0.44 | 14.02 | 4.64 | 2.02 | 13.99 | 13.75 |
|  | Test | Striatum | 0.25 | 13.09 | 7.75 | 0.69 | 0.26 | 11.92 | 4.35 | 1.74 | 11.31 | 12.85 |
|  | Retest |  | 0.54 | 15.01 | *NA* | *NA* | 0.58 | 15.06 | 4.64 | 2.25 | 14.96 | 14.93 |
|  | Test | Temporal Cortex | 0.23 | 12.30 | 7.16 | 0.72 | 0.24 | 11.23 | 4.35 | 1.58 | 10.58 | 11.99 |
|  | Retest |  | 0.45 | 13.97 | *NA* | *NA* | 0.48 | 14.06 | 4.64 | 2.03 | 13.96 | 13.90 |
|  | Test | Thalamus | 0.24 | 13.65 | 7.51 | 0.82 | 0.25 | 11.10 | 4.35 | 1.56 | 10.42 | 12.30 |
|  | Retest |  | 0.51 | 14.03 | 12.79 | *NA* | 0.54 | 13.92 | 4.64 | 2.00 | 13.85 | 14.10 |
|  | Test | White Matter | 0.19 | 11.61 | 6.00 | 0.94 | 0.20 | 10.47 | 4.35 | 1.41 | 9.44 | 11.20 |
|  | Retest |  | 0.49 | 12.82 | 1.66 | 6.72 | 0.44 | 12.79 | 4.64 | 1.76 | 12.63 | 12.93 |
| **NHP3** | Test | Cerebellum | 0.35 | 10.48 | 6.39 | *NA* | 0.33 | *NA* | 8.22 | *NA* | 9.24 | 10.32 |
|  | Retest |  | 0.26 | *NA* | 6.41 | *NA* | 0.24 | *NA* | 9.07 | *NA* | 9.16 | 11.31 |
|  | Test | Frontal Cortex | 0.53 | 19.94 | 13.17 | *NA* | 0.55 | 18.77 | 8.22 | 1.51 | 18.41 | 19.36 |
|  | Retest |  | 0.42 | *NA* | 12.79 | *NA* | 0.45 | 18.59 | 9.07 | 1.07 | 16.78 | 20.21 |
|  | Test | Parietal Cortex | 0.40 | 18.71 | 12.37 | *NA* | 0.41 | 17.78 | 8.22 | 1.38 | 17.46 | 18.19 |
|  | Retest |  | 0.32 | *NA* | 13.02 | *NA* | 0.33 | 18.28 | 9.07 | 1.04 | 16.57 | 19.72 |
|  | Test | Striatum | 0.51 | 17.60 | 12.01 | *NA* | 0.53 | 16.73 | 8.22 | 1.24 | 16.41 | 17.23 |
|  | Retest |  | 0.44 | *NA* | 11.34 | *NA* | 0.46 | 17.64 | 9.07 | 0.97 | 15.52 | 18.31 |
|  | Test | Temporal Cortex | 0.37 | 15.50 | 8.97 | *NA* | 0.37 | 14.95 | 8.22 | 1.00 | 14.25 | 15.12 |
|  | Retest |  | 0.29 | *NA* | 9.63 | *NA* | 0.30 | *NA* | 9.07 | *NA* | 13.50 | 16.34 |
|  | Test | Thalamus | 0.45 | 17.60 | 12.45 | *NA* | 0.48 | 16.27 | 8.22 | 1.18 | 16.04 | 17.00 |
|  | Retest |  | 0.34 | *NA* | 11.34 | *NA* | 0.36 | 17.03 | 9.07 | 0.90 | 14.73 | 17.41 |
|  | Test | White Matter | 0.41 | 16.36 | 9.76 | *NA* | 0.42 | 15.61 | 8.22 | 1.09 | 14.88 | 15.99 |
|  | Retest |  | 0.32 | *NA* | 10.04 | *NA* | 0.32 | 18.23 | 9.07 | *NA* | 13.80 | 16.97 |
| **NHP4** | Test | Cerebellum | 0.39 | *NA* | 10.81 | *NA* | 0.39 | *NA* | 10.99 | *NA* | 12.07 | 13.27 |
|  | Retest |  | 0.34 | *NA* | 10.80 | *NA* | 0.34 | 18.89 | 10.70 | 0.80 | 11.41 | 11.82 |
|  | Test | Frontal Cortex | 0.38 | *NA* | 10.76 | *NA* | 0.38 | *NA* | 10.99 | *NA* | 12.04 | 13.14 |
|  | Retest |  | 0.31 | 12.14 | 10.20 | *NA* | 0.31 | 12.71 | 10.70 | *NA* | 11.35 | 11.72 |
|  | Test | Parietal Cortex | 0.41 | *NA* | 12.79 | *NA* | 0.42 | 15.03 | 10.99 | 0.37 | 14.10 | 15.28 |
|  | Retest |  | 0.38 | *NA* | 14.99 | *NA* | 0.38 | 18.66 | 10.70 | 0.78 | 15.57 | 15.91 |
|  | Test | Striatum | 0.63 | *NA* | 15.06 | *NA* | 0.63 | 16.50 | 10.99 | 0.50 | 16.40 | 17.97 |
|  | Retest |  | 0.46 | *NA* | 15.36 | *NA* | 0.47 | 15.26 | 10.70 | 0.45 | 15.75 | 16.14 |
|  | Test | Temporal Cortex | 0.44 | *NA* | 12.30 | *NA* | 0.44 | 15.47 | 10.99 | 0.41 | 13.95 | 15.35 |
|  | Retest |  | 0.36 | 13.95 | 11.78 | *NA* | 0.36 | 13.53 | 10.70 | 0.29 | 13.13 | 13.54 |
|  | Test | Thalamus | 0.53 | *NA* | 12.69 | *NA* | 0.54 | 15.12 | 10.99 | 0.38 | 14.02 | 15.59 |
|  | Retest |  | 0.38 | *NA* | 12.88 | *NA* | 0.38 | 13.98 | 10.70 | 0.33 | 13.28 | 13.62 |
|  | Test | White Matter | 0.41 | *NA* | 11.24 | *NA* | 0.42 | *NA* | 10.99 | *NA* | 12.88 | 14.37 |
|  | Retest |  | 0.35 | 14.29 | 11.74 | *NA* | 0.35 | 13.35 | 10.70 | 0.27 | 12.91 | 13.41 |

*Abbreviations: NA = undetermined (%SE>25%)*

Supplementary Figure 1:*
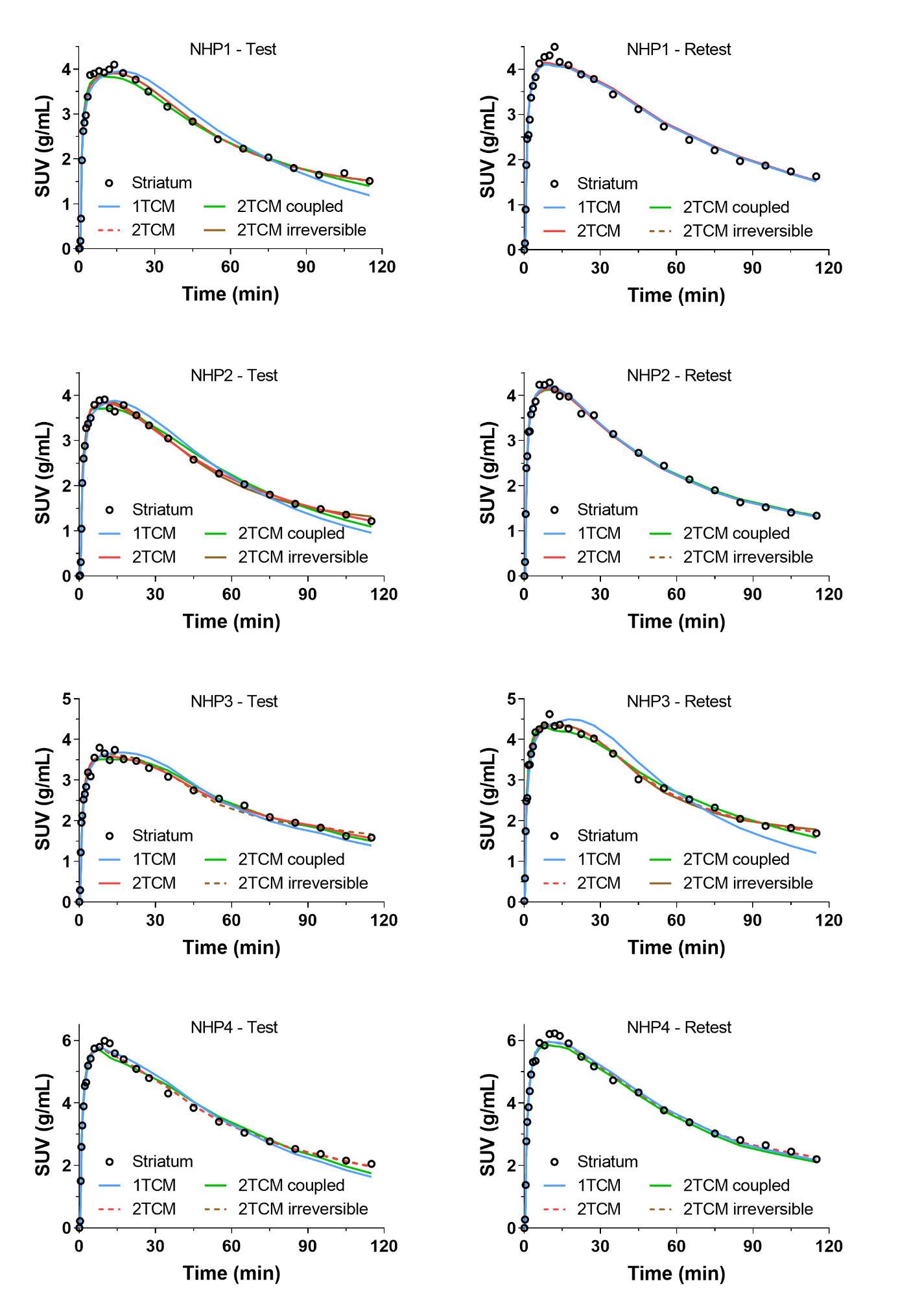
*

Individual fits for striatal region are shown for each test, and retest study for each individual NHP. When V_T_ did not converge using 2TCM (dashed red line, %SE>25%, 48% of the data), k_4_ tended to zero in 70% of these cases (less than 30% of all data). Even though the dose-dependent displacement of [^18^F]UCB-H by [^19^F]UCB-H and LEV, clearly supports the reversible binding kinetics of [^18^F]UCB-H to SV2A, we have considered the use irreversible models/approaches to quantify our data set, particularly using a 2TCM irreversible model. Briefly, AIC criteria was comparable between 2TCM-irr and 2TCM, but using 2TCM-irr λk3 and KI converged in 48% of all cases (%SE<25%), and for none of these parameters ICC or aTRV could be calculated (dashed brown lines if 2TCM-irr did *not* converge, %SE>25%). Based on these results, we concluded that the irreversible 2TCM fit of [^18^F]UCB-H data does not provide an accurate fit of the data.
